# Simulation of photic effects after cataract surgery for off-axis light sources

**DOI:** 10.1371/journal.pone.0262457

**Published:** 2022-01-20

**Authors:** Pooria Omidi, Alan Cayless, Achim Langenbucher

**Affiliations:** 1 Department of Experimental Ophthalmology, Saarland University, Homburg, Saarland, Germany; 2 School of Physical Sciences, The Open University, Milton Keynes, United Kingdom; University of Toronto, CANADA

## Abstract

Photopsia is a phenomenon that sometimes disturbs patients after cataract surgery. To evaluate the impact of the edge design of intraocular lenses (IOL) on the location, shape and relative intensity of photic effects at the retina caused by photopsia in pseudophakic eyes, photopsia was simulated using ZEMAX software. The structural parameters of the pseudophakic eye model are based on the Liou-Brennan eye model parameters with a pupil diameter of 4.5 mm. The IOLs implanted in the eye model have a power of 21 diopter (D) with optical diameter of 6 mm and 7 mm. From the ray-tracing analysis, covering variations of incident ray angle of 50° to 90° from temporally, a photic image is detected at the fovea at specific ray angles of 77.5° (6 mm IOL) and 78.2° (7 mm IOL). This photic image disappears when a thin IOL with an edge thickness of 0 mm or a thick IOL with absorbing edges is replaced in the eye model. With an anti-reflective edge, this photic image remains, but with a fully reflecting edge it disappears at the critical angles and appears with different shapes at other angles. The intensity of this photic image can be reduced by changing the edge design to a frosted surface. Most of the photic patterns in IOLs are not observed with absorbing and thin edge designs. IOLs with anti-reflecting and fully reflecting edges generate disturbing photic effects at different angles on the fovea. IOLs with frosted edges reduce the contrast of the photic effects and make them less disturbing for patients.

## Introduction

Photopsia is an optical phenomenon defined by the presence of effects in the visual field which are not directly correlated to imaging of an object. In photopsia light is detected at a location where no light is expected.

This phenomenon sometimes occurs in a time period shortly after intraocular lens implantation. In the literature it is shown that this effect could be associated with the position and optical design of IOLs, as first reported by Masket et al. [1] The effect has been named *dysphotopsia* by Tester et al. [2].

Several explanations, such as: IOL material with a high index refractive index [3–5], optics with sharp or truncated edges [3–5], defects in the IOL optics during manufacturing, central optical defects during folding and injection, a cataract incision located temporally in the clear cornea [6], a prominent globe [7], a shallow orbit [7], neural adaptation [8], and reflection of the anterior capsulotomy edge projected onto the nasal peripheral retina for photopsia, have been discussed as effect sizes [9]. Holladay et al. determined the impact of the edge design as the cause of this phenomenon using a ZEMAX program simulation [10, 11].

Photopsia cannot be measured or quantified objectively and there is no clinical test to prove or verify these phenomena. However, in rare cases, it can be detected with a 90° visual field measurement. Generally, following cataract surgery, patients are either free of these photic side effects or the effects are below perceptible levels. A few cases who encounter these symptoms can tolerate them and not feel disturbed [3, 12]. Some patients report symptoms with the help of drawings originated from photopsia.

In photopsia light is perceived as bright patterns such as arcs [10], streaks [13], rings or halos [14] which become visible on the central or mid peripheral visual field without correlation to illumination. In contrast, in some cases, this phenomenon causes a gap in the homogeneous lighting of the retina that appears as a crescent, sickle, arc, or ring shadows [3].

Clinical studies show that photopsia always appears shortly after cataract surgery, that the photic effects are mostly located in the temporal visual field, and that they can be made to disappear with the use of ocular blinkers. Moreover, there is no systematic correlation between photopsia and corneal diameter, anterior chamber depth, iris pigmentation, and photopic and scotopic pupil diameter or refraction [6, 14].

## Methods

This study was registered at the local ethics committee of the Medical Association of Saarland (Ärztekammer des Saarlandes, 157/21).

### Eye model specifications

The pseudophakic eye model used for optical simulations in this study is based on the Liou-Brennan schematic model eye introduced in 1997 [15]. The cornea of this model is defined by two aspheric refracting surfaces with a thickness of 0.5 mm. These two rotationally symmetric surfaces (front and back surface) are described by a radius of 7.77 and 6.40 mm, an asphericity of -0.18 and -0.60, and a refractive index of 1.376 respectively. Other parameters of this model are as follows: internal ACD, 3.79 mm; decentration of the pupil nasally, 0.5 mm; distance between pupil and IOL, 0.63 mm; total axial length, 23.95 mm. The model is centred on the optical axis and therefore, the retinal surface is symmetrical about the optical axis. Moreover, the pupil diameter used in this model is 4.5 mm.

**Fig 1:**
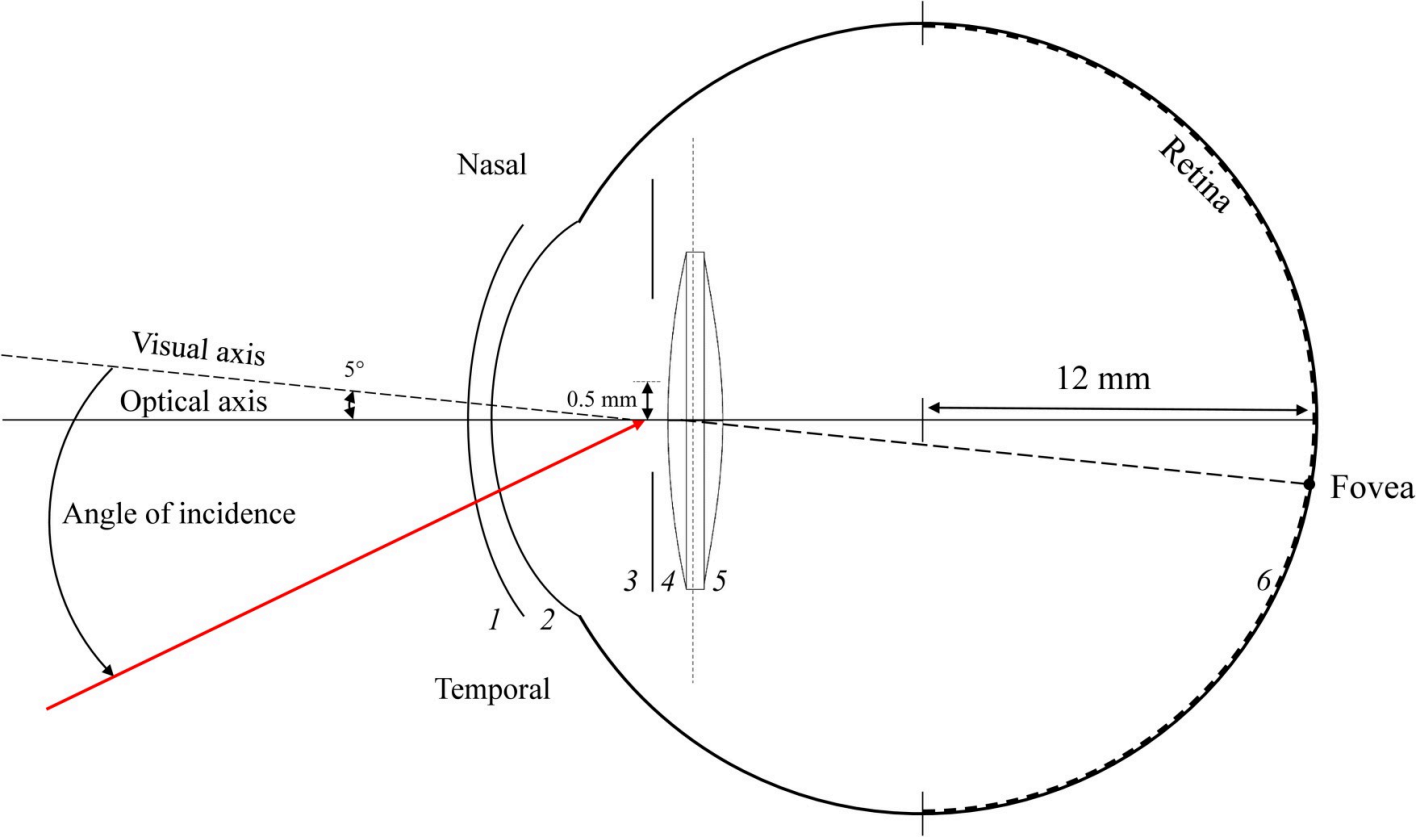
Schematic drawing of the pseudophakic eye. (See Table 1 for parameter values).

Fig 1 illustrates a schematic sketch of the eye model used in this study. Each surface is labelled with a number from 1 to 6.

The IOLs in this model are acrylic hydrophilic with refractive power of 21 dpt and refractive index of 1.458, an edge thickness of 0.3 mm and a sharp circular optic edge. The front surface of the IOLs defined by a spherical surface, but the back surface is an aspherical surface optimised for 0.26 μm spherical aberration in the total eye model. The optical diameters of the IOLs were 6 mm and 7 mm, respectively. The lenses were positioned with the equator aligned to the equator of the crystalline lens in the model eye. Table 1 lists all relevant parameters of the proposed pseudophakic model eye and the polynomial coefficients that describe the rotationally symmetric aspheric surface of the IOL. Note that polynomial coefficient values not mentioned in the table equal zero.

**Table 1:** Structural parameters of the pseudophakic eye model.

Surface	Radius[mm]	Asphericity	Thickness[mm]	Optical diameter[mm]
1	7.77	-0.18	0.5	14
2	6.40	-0.6	3.16	14
3	∞	-	0.63	4.5
4	13.86	-	0.97	6 and 7
5	-11.66	-1.5	18.69	6 and 7
6	-12	-	-	24
Surface	α_2_[mm^-1^]	α_4_[mm^-3^]	α_6_[mm^-5^]	α_8_[mm^-7^]
5	-6.34e^-3^	1.15e^-3^	-3.86e^-7^	-2.47e^-8^

### Ray tracing procedure

Ray tracing was performed with the ZEMAX professional ray tracing software (Version 19.8, Washington, USA) using the non-sequential ray tracing mode to study the photopsia. For this simulation, an extended light source with a diameter of 6 mm was used.

To identify the position of the fovea on the retinal surface, rays are traced through the pupil with a physiological entrance angle slanted by 5° with respect to the optical axis in the nasal direction. The retina is modelled as a half-sphere with a radius of 12 mm.

A collimated light pencil made up of 1 million rays from the extended source in the visible range is used to analyse the photopsia. Rays from 50 to 90 degrees in steps of 5° with respect to the optical axis are traced temporally through the pupil to evaluate the intensity distribution on the retinal surface.

At some specific angles between 50° to 90°, arc-shaped ghost images in the foveal region were noticed. For these specific angles further simulations were performed.

By simulating a thick absorbing IOL edge, light transmission through and reflection off the edge is suppressed. With thick specular reflecting and anti-reflecting IOL edges, the effects of internal specular reflection and transmission are highlighted respectively. With a thick scattering IOL edge, the effect of ‘frosting’ is resampled and the transmitted light is scattered, as is the internally reflected light. The Lambertian scattering model is used to define the frosted edge surface in this IOL type. To avoid complexity, an isotropic scattering model was applied, in which the scattered ray projection vector has an equal probability for all directions in the space. Lambertian scattering is independent of the incident ray angle and each point on the surface was treated as a point source.

In contrast, with the thin optics edge no light transmission or internal light reflection occurs at the edge. These settings enabled the impact of transmission, reflection or scattering of the optical edge on the intensity distribution in the retina to be studied.

In summary, to evaluate the contribution of the IOL edges on photopsia, IOLs were simulated with thin edges (0.0 mm instead of 0.3 mm), and additionally with absorbing, fully reflecting, anti-reflecting, and scattering edges (0.3 mm edge).

## Results

**Fig 2:**
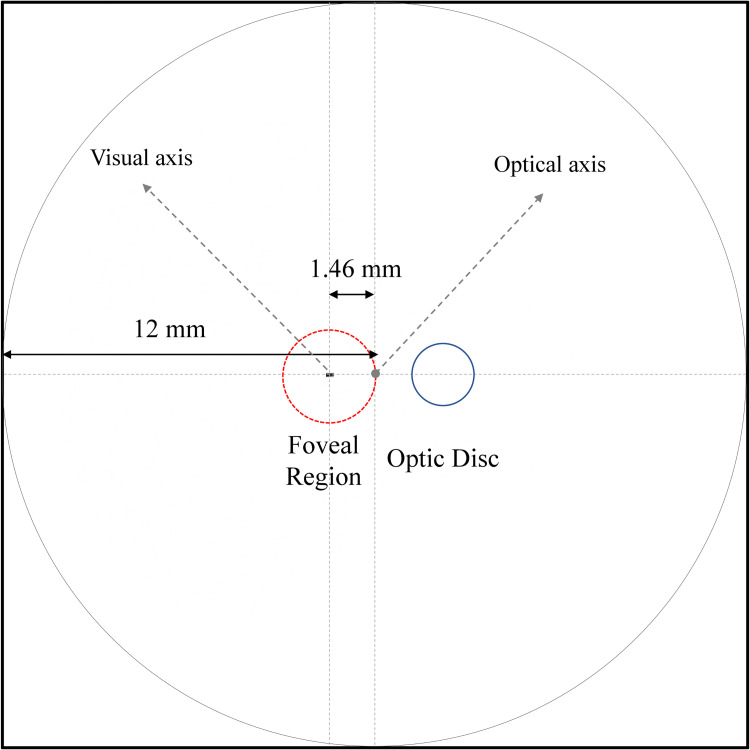
Fovea and optic disc positions on the retinal surface.

Fig 2 shows the simulation results from the foveal position on the retinal surface when the entrance angle is 0° with respect to the visual axis. The simulation results show that the foveal position is not located at the geometrical axis. It is shifted 1.462 mm towards temporally.

**Fig 3:**
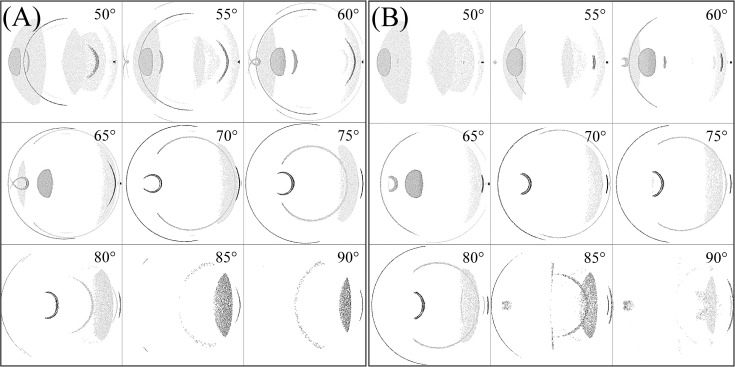
Intensity distribution on the retina for variation of incident ray angle of 50° to 90°. (A) For IOL with 6 mm optics diameter. (B) For IOL with 7 mm optics diameter.

Fig 3 shows the light intensity profile on the retina for the IOL types (6 mm and 7 mm optics) for variations of incident ray angle of 50° to 90° temporally in steps of 5°.

**Fig 4:**
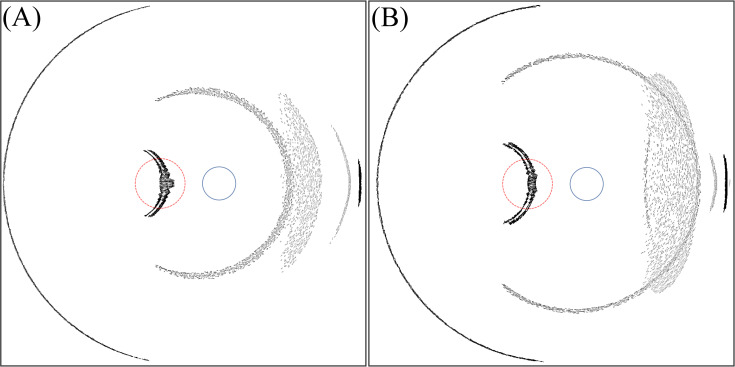
Intensity distribution on the retina for IOL with the standard edge design. (A) For angle of incidence of 77.5° and 6 mm optics diameter. (B) For angle of incidence of 78.2° and 7 mm optics diameter.

**Fig 5:**
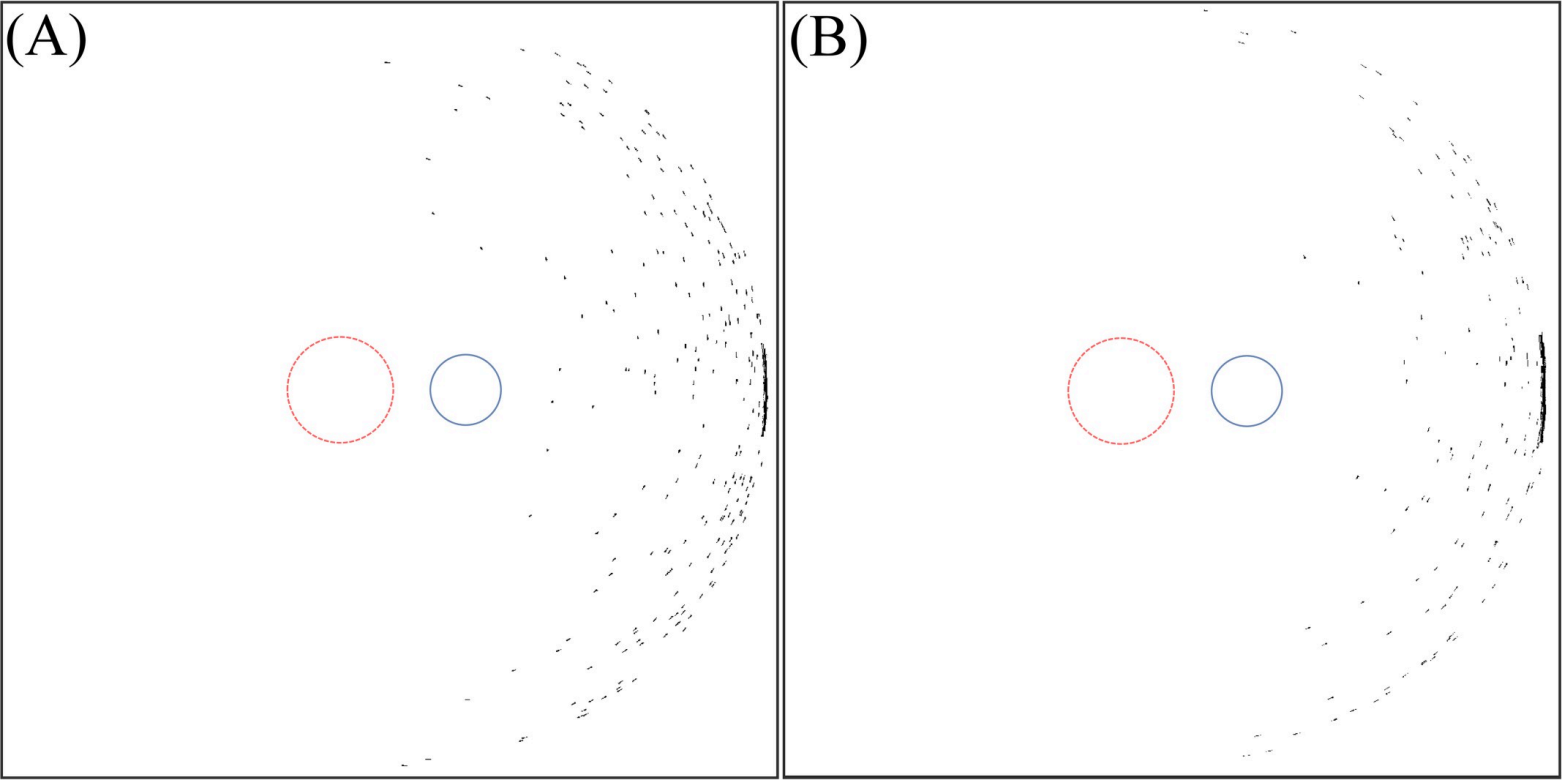
Intensity distribution on the retina for IOL with absorbing edge design. (A) For angle of incidence of 77.5° and 6 mm optics diameter. (B) For angle of incidence of 78.2° and 7 mm optics diameter.

**Fig 6:**
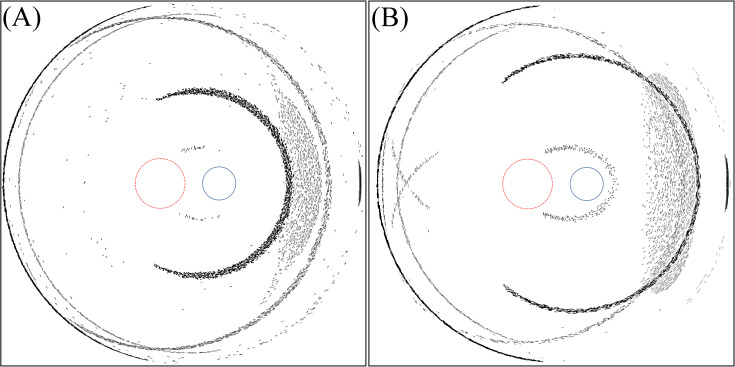
Intensity distribution on the retina for IOL with fully reflecting edge design. (A) For angle of incidence of 77.5° and 6 mm optics diameter. (B) For angle of incidence of 78.2° and 7 mm optics diameter.

**Fig 7:**
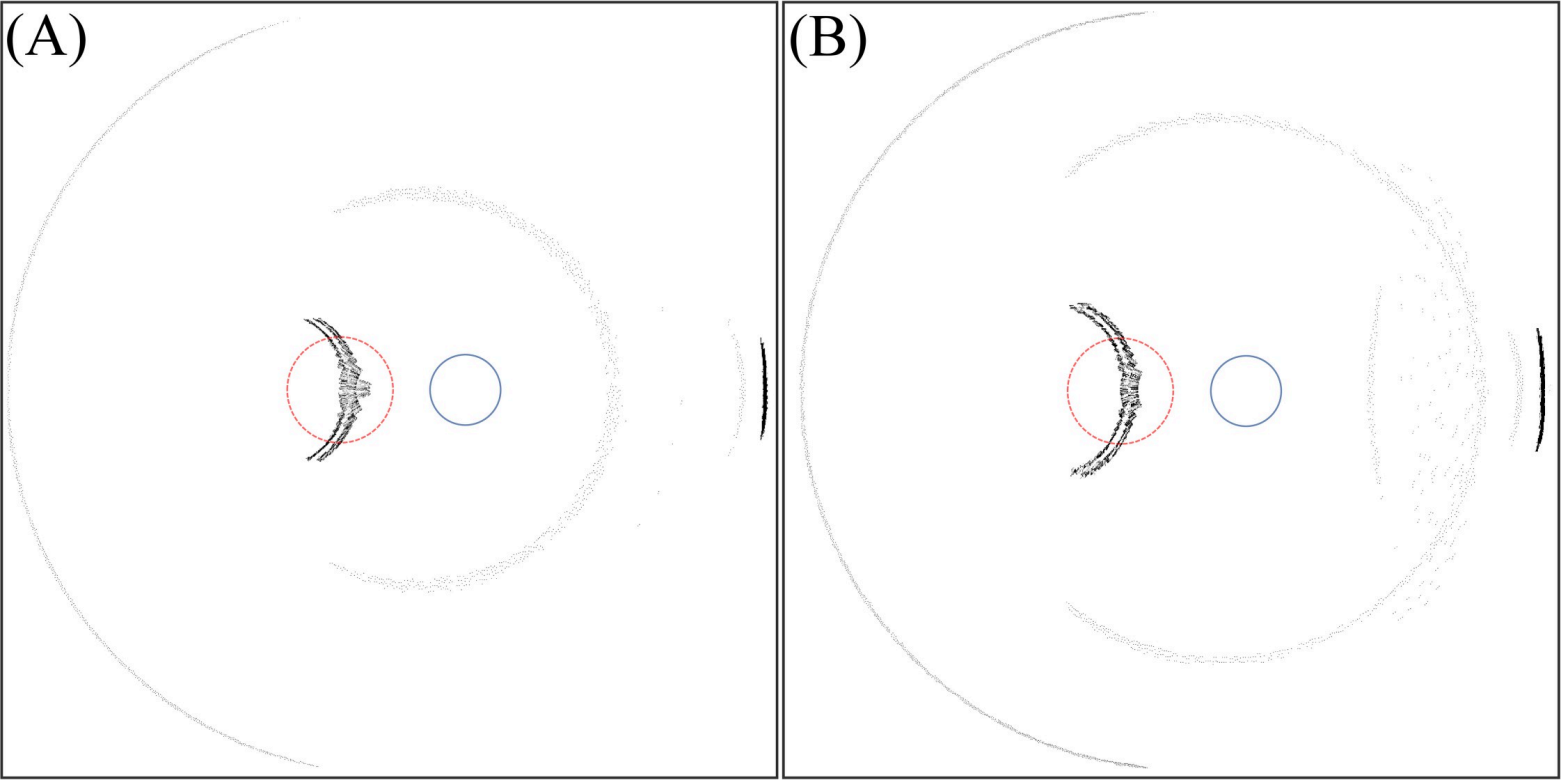
Intensity distribution on the retina for IOL with anti-reflecting edge design. (A) For angle of incidence of 77.5° and 6 mm optics diameter. (B) For angle of incidence of 78.2° and 7 mm optics diameter.

**Fig 8:**
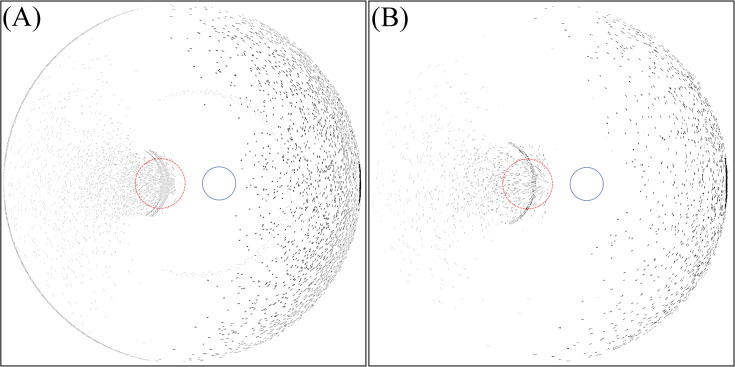
Intensity distribution on the retina for IOL with frosted edge design. (A) For angle of incidence of 77.5° and 6 mm optics diameter. (B) For angle of incidence of 78.2° and 7 mm optics diameter.

**Fig 9:**
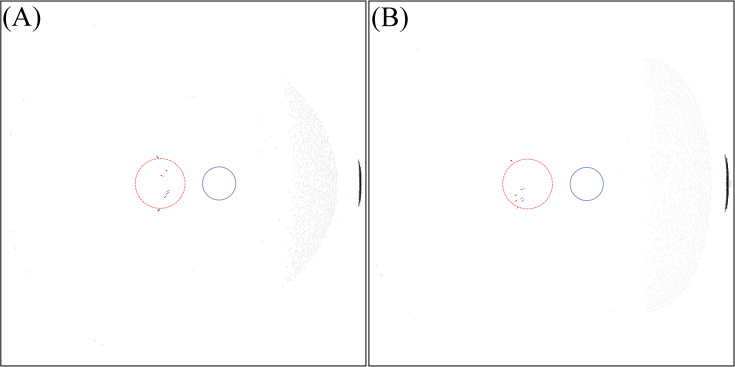
Intensity distribution on the retina for IOL with thin edge design. (A) For angle of incidence of 77.5° and 6 mm optics diameter. (B) For angle of incidence of 78.2° and 7 mm optics diameter.

Within this variation of the incident ray angle, we note a specific incident ray angle of 77.5° (6 mm optics) and 78.2° (7 mm optics) where an arc-shaped photic image is located at the fovea. Fig 4 shows the light intensity distribution at these specific angles. Note that in Figs 3–9, section (A) refers to the 6 mm optic diameter with incidence angle of 78.2° and section (B) refers to the 7 mm optic diameter with incidence angle of 77.5°. Moreover, the red circle with a dashed line illustrates the foveal region and the smaller circle with a solid line illustrates the optic disc.

In a further analysis we analysed how the different edge conditions affect the properties of this photic phenomenon. Fig 5 illustrates the intensity distribution at the retina for IOLs with absorbing edges.

By changing the edge type between anti-reflective and fully reflective surfaces, photic patterns generated by transmission or reflection of light from the edges can be classified independently. Anti-reflective edges remove the photic patterns that arise from internal reflection at the edges. On the other hand, fully reflective edges increase the intensity of the photic patterns caused by internal reflection while eliminating light passing through the edges. Figs 6 and 7 show the intensity distribution in situations where the edges are fully reflective and anti-reflective respectively.

Fig 8 illustrates the intensity distribution at the retina with a frosted IOL optics edge design.

In the final ray tracing simulation, the contribution of edge thickness on different photic patterns is analysed. Fig 9 illustrates the retinal image using a thin edge design (0 instead of 0.3 mm) which fully avoids edge effects such as transmission or internal reflections.

**Fig 10:**
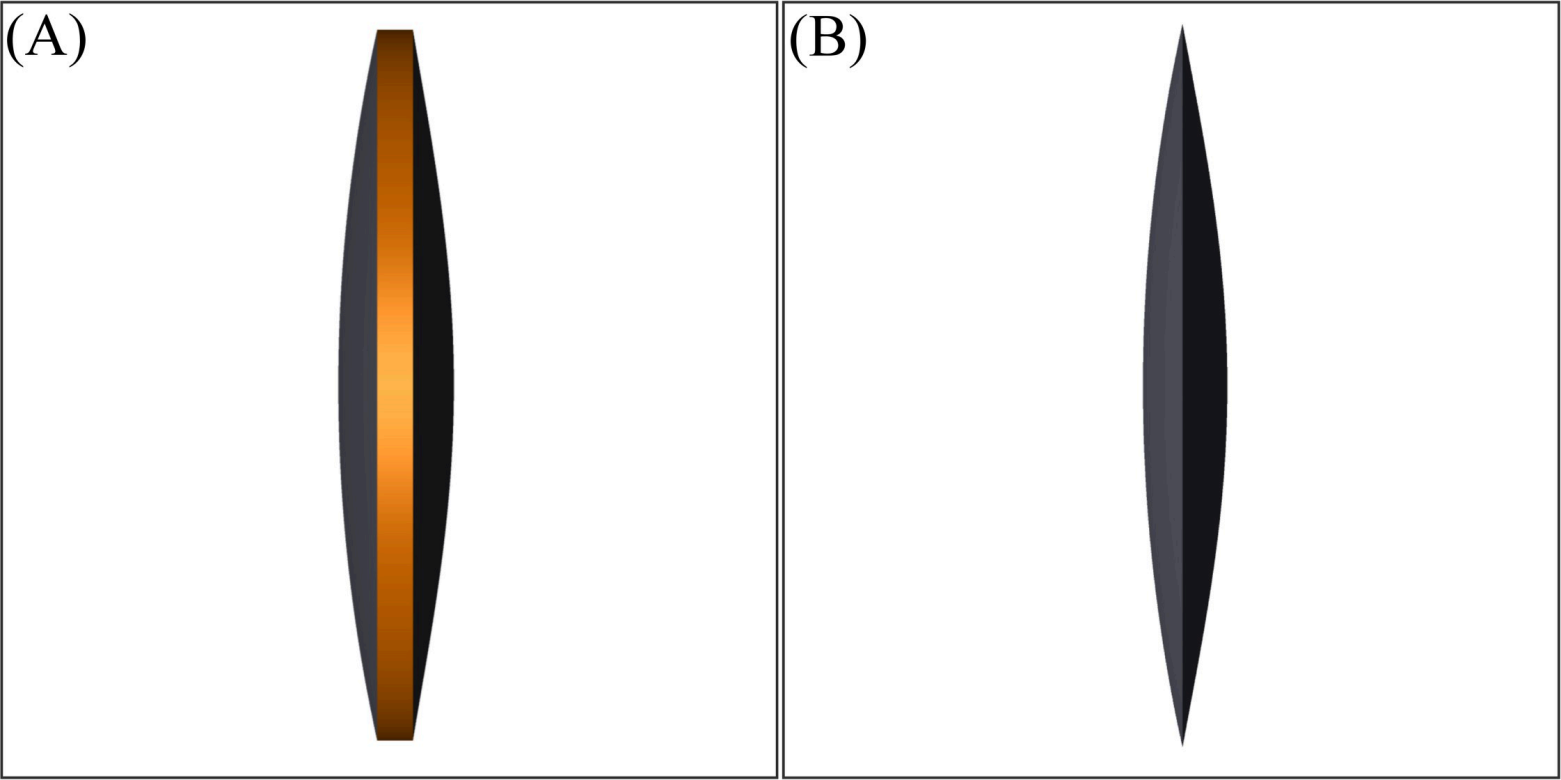
Schematic drawing of the simulated IOLs. (A) Thick IOL with edge thickness of 0.3 mm. (B) Thin IOL with edge thickness of 0.0 mm.

Fig 10 illustrates a schematic view of the thick and thin IOLs in this study. In 10A the colored part shows the edge surface is and in 10B edge thickness is zero.

## Discussion

From Fig 3 it is observed that with increasing incident ray angle photic patterns initially located far nasally move to the central part of the retina and change their shape and intensity. Similarly, ellipse-like shapes with a lower intensity located at the centre move temporally at the retina. From 50° to 65°, a bean shaped pattern is detected nasally. Moreover, a crescent shape pattern is observed for incident ray angles between 60° and 80° changing in intensity, size, and shape with variation of the incident angle.

With a 6 mm optics IOL, light passing through the interspace between iris and lens is observed from 50° in the temporal region. In observing this effect in IOLs with a 7 mm optics diameter, the inclination angle is 55° as a result of the narrower channel between iris and IOL. In both IOL types, with increasing angle of incidence, the crescent shape pattern moves towards the periphery. The intensity of this crescent increases up to angle of 75° and then decreases. Finally, it can be concluded that between 65° and 80°, photic patterns are located in the foveal region.

From Fig 4, at a ‘critical’ incident ray angle this light effect hits the fovea, where we expect a serious optical disturbance of the patient because light sensitivity is very high in the foveal spot (Fig 2).

As expected, Fig 5 shows that most of the photic patterns disappear with absorbing an edge.

**Fig 11:**
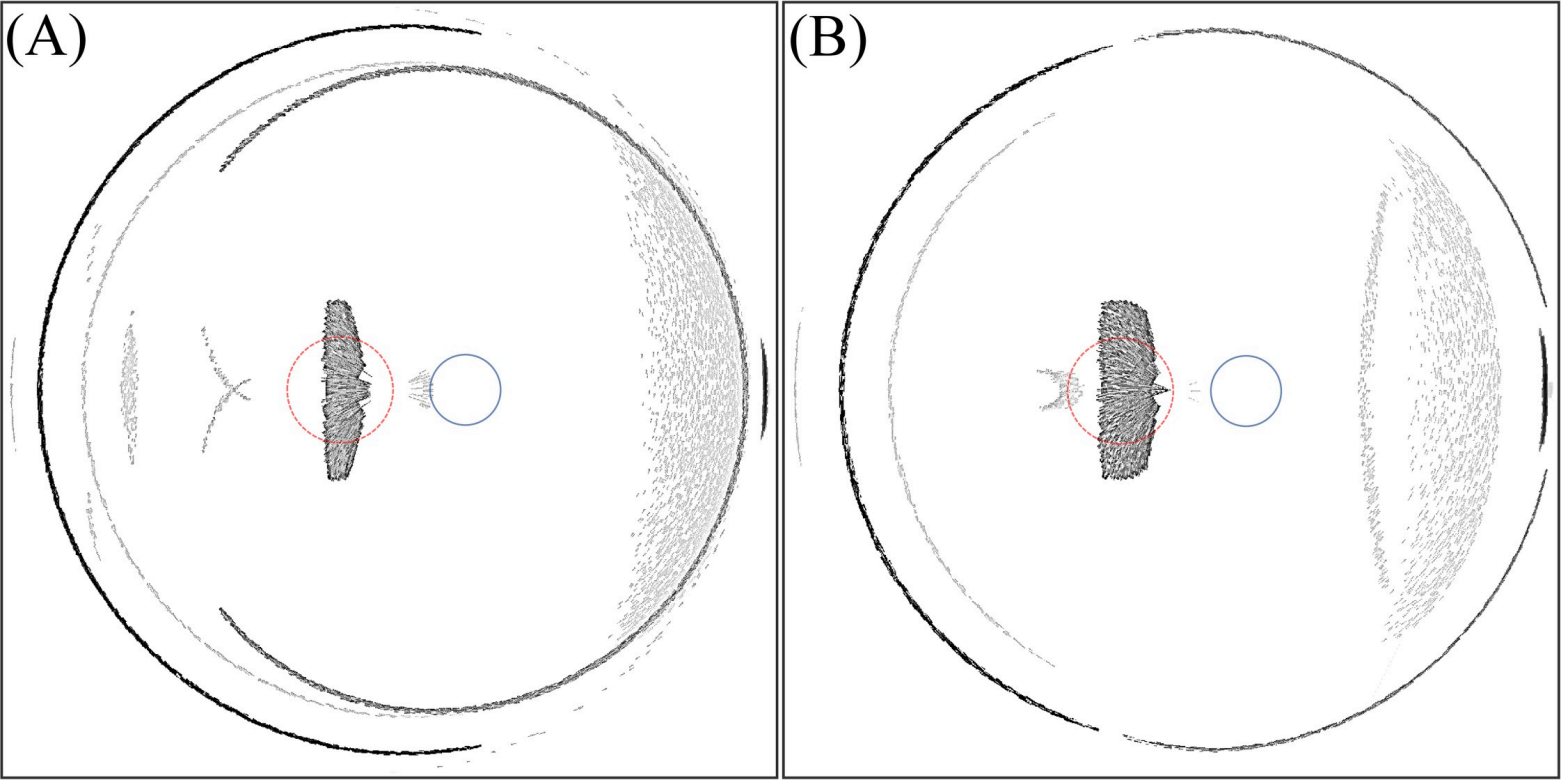
Intensity distribution on the retina for IOL with thin edge design. (A) For angle of incidence of 67.4° and 6 mm optics diameter. (B) For angle of incidence of 68.8° and 7 mm optics diameter.

By analysing Figs 6 and 7, it is concluded that photic patterns can be caused by reflection or transmission of light through the edge of the IOL. In Fig 6, a fully reflective edge causes the crescent that is located at foveal region. Simultaneously, this edge type enhances the intensity of the larger crescent on the temporal region of the retina. Within the temporal region far from the fovea, the sensitivity of the retina is lower and therefore serious optical disturbance is not expected, but the results show that the photic effect could appear in different retinal regions depending on the incident ray angle. The photic effect caused by internal reflection at the edge can appear on the fovea when the incidence angle is 67.4° and 68.8° for IOLs with 6 mm and 7 mm optics diameter respectively. Fig 11 shows the light intensity distribution for this situation.

On the other hand, the anti-reflective edge causes the large crescent in the temporal region to disappear but the smaller crescent located on the foveal region is still present and could again cause serious optical disturbance because it is located at the fovea.

Variously, the frosted optics edge design causes a particular effect on the photic patterns on the retinal surface. From Fig 8, it is concluded that the frosted edge scatters the rays transmitted or internally reflected from the IOL edge.

Technically, this effect does not change the overall intensity of the light transmitted or reflected by the edge, but it distributes the light intensity over a larger retinal area, thus reducing the contrast of the patterns. As a result, patients will have less optical disturbance with IOLs with frosted edges by reducing the contrast of the photic effects.

It should be noted that in all of the results with different edge types, the temporal photic pattern generated by light passing through the interspace between iris and IOL does not change. Consequently, modifying the edge design does not affect this aspect of the photic effect. Although it is not possible to eliminate this photic effect with IOL customisation, it does not disturb patients, being located away from the fovea as the most sensitive region of the retina.

Finally, by studying Fig 9, it is observed that there is no photic pattern in the nasal region of the retina with the thin edge design. As expected, the photic pattern originated by the light passing through the interspace between iris and lens, is also observed with this thin edge IOL.

## Supporting information

S1 Table (XLSX)
